# The general impact of self-stigma of mental illness on adult patients with depressive disorders: a systematic review

**DOI:** 10.1186/s12912-024-02047-z

**Published:** 2024-06-25

**Authors:** Refah Alqahtani, Alan Pringle

**Affiliations:** 1 Faculty of Nursing and Applied Medical Sciences, College of Applied Medical Science in Jubail, Imam Abdulrahman Bin Faisal University (IAU), Jubail Industrial City, Saudi Arabia; 2 Faculty of Medicine & Health Sciences, School of Health Sciences, Nottingham University, Nottingham City, UK

**Keywords:** Stigma, Self-stigma, Mental Illness, Depression

## Abstract

**Background:**

Mental illness stigma is often common among mentally ill patients. This stigma can come from others or the patients themselves, which is called ‘self-stigma’. The present study explored the widespread impacts of self-stigma on adult patients with depression. Additionally, this review compared the severity of self-stigma levels among psychiatric disorders and to review and update thoughts about self-stigma of depression.

**Methods:**

An etiology and risk systematic review was conducted using the Joanna Briggs Institute (JBI) approach as a guideline. The search process was performed via research databases including MEDLINE, EMBASE and CINAHL. The inclusion criteria are studies include participants diagnosed with depressive disorders, both genders, participants’ exposure to mental illness self-stigma, participants’ experience of self-stigma consequences and any geographical site or clinical settings are included, the type of the included studies must be observational studies. The included studies were limited to the English language studies that were published from 2016 and onwards. Patients with depression under the age of eighteen and patients diagnosed with multiple mental illnesses were excluded. The JBI critical appraisal checklist were adopted to assess the risk of bias.

**Results:**

In December 2022, a comprehensive search yielded eight cross-sectional studies that were included in this systematic review, involving a total of 783 patients diagnosed with depression, and 28 studies were excluded for not fulfilling the inclusion criteria of the review. The findings were extracted and synthesized through textual narrative synthesis into three main categories negatively affected by self-stigma of depression. These are: (1) the impact on the quality of life, (2) the impact on self-esteem and (3) the impact on self-worth. Moreover, in regard to the comparison of self-stigma levels among psychiatric disorders, self-stigma for people with schizophrenia was higher than self-stigma of depression.

**Conclusion:**

Self-stigma of depression has negatively impacted multiple aspects of the patient’s life. Thus, the review brings the following recommendations: increase community awareness, educate the healthcare providers, include the topic of mental illness stigma in academic curriculums. The main limitation of the review is the limited number of included studies.

**Trial registration:**

The research proposal for this review has been registered to Prospero (ID number: CRD42022366555).

**Supplementary Information:**

The online version contains supplementary material available at 10.1186/s12912-024-02047-z.

## Background

Depression is a mood disorder in which people exhibit constant feelings of sadness, decreased pleasure and a loss of daily functioning [1]. Depression affects about 3.8% of the world’s population [2]. Furthermore, it is the leading cause of suicide [3]. For instance, the United States reported that 40% to 80% of adults who attempted to commit suicide were found to have been diagnosed with depressive disorder [4]. The global rate of depression among adults is estimated to be 10.7% [2]. The mental and psychological changes often appear in adulthood [5]. Consequently, the peak age of onset for mental illnesses occurs at this time [6]. Thus, adult patients with depressive disorders become more susceptible to stigma [7], which increases their risk of experiencing the negative effects of mental illness stigma [8]. Depression is strongly associated with self-stigma. The prevalence of self-stigma among patients with depression revealed a significant rate of 29% [9]. The self-stigma, however, is discussed more with schizophrenic disorder rather than mood disorders such as depression. Patients with schizophrenia suffer more intensely and regularly from self-stigma than patients with other mental illnesses [10]. Additionally, the level of self-stigmatization is altered among psychiatric disorders.

### Mental illness stigma

Mental illness stigma (MIS) is defined as a negative combination of biases, discrimination and stereotypes towards people diagnosed with mental illness [11]. The MIS produces daily life challenges to psychiatric patients. These challenges could include negative attitudes such as negative self-perceptions and negative public behaviors [12]. MIS is divided into four forms: personal, public, perceived and self-stigma. First, personal stigma is a person’s opinions, feelings and manners towards individuals with mental disabilities [8]. Second, public stigma refers to negative beliefs and attitudes from the general public towards patients with mental illness [13]. Third, associated with public stigma is perceived stigma, which refers to the person’s view and concern of others’ reactions towards individuals with mental disabilities [8]. Fourth, self-stigma which is also called ‘internalized stigma’, is described by the patient’s feeling of shame, low self-esteem and embarrassment towards having a mental and psychological problem [14]. Furthermore, A study highlighted that once psychiatric patients acknowledge that they need to seek mental healthcare services, this might lower their self-esteem, which consequently forms their self-stigma [14]. Therefore, self-stigma will disturb the occupational and social life of the patients [14–16]. Moreover, the self-stigma of depression (SSD) has been associated with the interruption of quality of life (QoL), suicidal ideation and reduced professional and social roles in the patient’s life [17]. Limited studies, however, have directly focused on the SSD [17]. In contrast, public stigma is studied more because it reflects the judgment and discrimination that the general population has toward individuals with mental illness [18]. Thus, public stigma is the most researched and studied type of stigma. The MIS has transformed over the ages. For instance, in 1955, 1956, and 1976, documents show a lack of public understanding about psychiatric disorders and a refusal to discuss cases of mental illness [19]. Mental illness stigma, however, decreased and the seeking of mental healthcare services increased between 1996 and 2006 [19]. Moreover, a recent displayed a remarkable reduction in public stigma towards depression [20]. The published research in the field of self-stigma of depression is still limited to studies concerning testing, examining the scales of self-stigma and studying the relationship between depression and self- stigma. Few of the published studies have addressed the impact of self-stigma on patients with depression. As mentioned earlier, public stigma is more researched than self-stigma, and self- stigma is discussed more in patients with schizophrenia than in patients with depression. Based on the searching process using the systematic reviews’ databases, the subject of the impact of self-stigma on adult patients with depression was barely covered and discussed. The authors of this review, however, found some studies focused on the effect of self-stigma on mental illnesses in general without specifying a particular mental illness, such as depressive disorders. Therefore, this review considered investigating the impact of SSD on adult patients. Further, there is an overlap between depression symptoms and self-stigma effects [20]. Therefore, the systematic reviews must be conducted by including studies that use reliable scales to measure the SSD, in order to differentiate between symptoms of depression and outcomes of self- stigma. Thus, this review intended to study the impact of self-stigma on participants who were identified as having SSD.

As mentioned earlier, since the stigma has changed over the ages, the need to conduct a reviews about MIS is needed. Therefore, the regular producing and publication of studies about mental illness stigma are required to cope with the changes in MIS. Hence, a search of the systematic reviews’ databases was conducted. This search showed several research papers concerning MIS in general, but limited research focused on the effect of self-stigma on patients with depressive disorders. Thus, there was a noticeable lack of published systematic reviews explicitly concentrated on identifying the impact of self-stigma on adult patients with depression. The current study’s purpose is to answer the following research question ‘what is the general impact of self-stigma on adult patients with depressive disorders?’. In addition to fulfilling the discussed gaps in the background with three intentions: (1) to identify the impact of self-stigma on patients with depressive disorders, (2) to review and update thoughts about self-stigma of depression, (3) to compare the level of depression self-stigma to the self- stigma of other common mental illnesses in order to recognize to what extent self-stigma effects depression.

## Methods

The systematic reviews for etiology and/or risk is utilized for this review. This kind of review is established to determine the relationship between specific exposure or risks and outcomes [21]. Furthermore, this type of review is used to determine the extent and impact between an exposure and a health outcome [22]. The research proposal for this review has been registered to Prospero (ID number: CRD42022366555). However, there is adjustment on the current review, that made it differ from the registered protocol. Researchers should consider modifying and expanding inclusion and exclusion criteria and minor changes to the research question after a deep understanding of the research topic [23]. This modification concerned the research question which has been slightly modified to become as follows: ‘the general impact of self-stigma on adult patients with depressive disorders’ instead of ‘the experience and impact of stigma in adult patients with depressive disorders’. The purpose for this change is to keep the research question focused on the impact only rather than experiences and to narrow the research question by specifying the type of stigma.

The traditional ‘PICO’ framework for systematic reviews of effectiveness does not align, however, with questions relating to risk and etiology [21]. A systematic review of etiology and risk should follow the ‘PEO’ framework, which indicates Population, Exposure of interest (independent variable) and Outcome (dependent variable) [21, 22]. The research question for this review is as follows: ‘What is the general impact of self-stigma on adult patients with depressive disorders’. Thus, the ‘PEO’ question framework is appropriately used as follow; P, Adult patients with depression disorders, E, Self-stigma of mental illness and O**,** General impact on the patients’ lives. The population category in this review is clearly stated as adult patients ages eighteen years and above who have been clinically diagnosed with one of the depressive disorders. The exposure is an independent variable, which is the patients’ exposure to self-stigma. The outcome of the present study is the general impact of self-stigma on adult patients with depression. The term ‘general impact’ includes many aspects in which self-stigma could affect patients’ lives, such as the quality of life and cognitive ability. The inclusion and exclusion criteria of the primary included studies are illustrated in Table 1.

**Table 1:** The inclusion and exclusion criteria of the primary included studies

**Item**	**Inclusion Criteria**	**Exclusion Criteria**
**Population**	Adult patients diagnosed with any depressive disorders. Both genders are included	Patients with depression under the age of eighteen. Patients diagnosed with multiple mental illnesses **Rationale:** This study aims to investigate the impact of self-stigma on patients with depression only. Therefore, any study that included participants diagnosed with depression besides other mental illness is excluded. Moreover, one of the review objectives is to compare the self-stigma of depression with other mental illnesses. In this case the participants for these studies will be separated and numbered, depending on each mental illness
**Exposure**	The patients’ exposure to self- stigma of depression	The patients’ exposure and experiences to other types of stigmas; personal, public and perceived stigma towards depression **Rationale:** This study is aimed to investigate the impact of one type of mental illness stigma, which is self-stigma on patients with depression
**Context**	This review will target global studies that explore SSD in any geographical site. The study’s settings are not limited. It will include psychiatric outpatient clinics, psychiatric in-patient wards, mental health rehabilitation and community settings	The settings and geographical sites are general. Therefore, there are no exclusion criteria regarding the context of this review
**Year of publication**	Studies were published in 2016 and onwards **Rationale:** The authors of this review included the research published in the last six years to cover the stigma trends in mental illness stigma	Studies were published from 2015 and earlier
**Studies Language**	Studies published in the English language only **Rationale:** Due to the shortage of time, since this review must be done within a specific timing	Studies published in other languages, rather than the English language
**Type of study**	Observational studies: these include cross-sectional, longitudinal, case – control, cohort studies, retrospective and prospective **Rationale:**Reviews of etiology and risk predominantly originate from observational studies [24].	Any study that is not an observational study

The search procedure initially started in February 2022 and ended in June 2022. The databases utilised were limited to: Medical Literature Analysis and Retrieval System Online (MEDLINE), Excerpta Medica Database (EMBASE) and Cumulative Index to Nursing and Allied Health Literature (CINAHL). The published and unpublished research that fulfilled the research criteria and that was related to the review topic was reviewed. The search included scanning the research results’ titles, abstracts. The suggested search strategy of the ‘three-phase process’ by the JBI was adopted for this review. First, an initial search was conducted in MEDLINE to identify all the possible keywords associated with the current research topic. Second, the previous step to all the included databases was applied with caution toward each specific database’s characteristic of searching methods. Third, the references list of the collected studies was scanned to find additional studies to prepare for the appraisal step. The authors of the current study examined the titles and abstracts of the search results and excluded research that obviously did not meet the inclusion criteria for this review. Full-text articles were retrieved and carefully inspected to ensure they contained the inclusion criteria. The methodological quality tool used to assess the primary studies for this review is the JBI critical appraisal checklist for analytical cross-sectional studies. Two independent reviewers appraised the quality of the selected studies. The JBI checklist was modified by removing two questions related to the confounding factors, since the included studies have not reported the presence of confounding. The studies were critically appraised based on the following criteria:

- Were the criteria for inclusion in the sample clearly defined?
- Were the study subjects and the setting described in detail?
- Was the exposure measured in a valid and reliable way?
- Were objective, standard criteria used for measurement of the condition?
- Were the outcomes measured in a valid and reliable way?
- Was appropriate statistical analysis used?

In this review, some of the included studies are dissimilar regarding participants and methods of assessing the exposure, which makes it inappropriate for the author to apply meta-analysis approach. Therefore, A textual narrative synthesis approach was utilised for this review. This approach aimed to collect the data based on categories derived from the studies’ findings [25]. This approach was selected intentionally as the research area has not been investigated before through a systematic review. Furthermore, narrative synthesis assists in searching systematically and classifying the data for the first time [26]. Moreover, according to the JBI framework the textual combination of data is recommended when the included studies are diverse in terms of population, methods, or findings [25]. Furthermore, any clarification or missing data regarding the included studies was resolved by contacting the authors. For instance, the authors of one of the included studies (Hasan and Musleh, 2018) [27] were contacted to seek clarification about the tool they used to assess self-stigma, because this will assist the authors of this review to properly evaluating and critiquing the paper. Lastly, the steps of this review have been reported through PRISMA 2020 checklists for abstract and main text.

## Results

**Fig. 1:**
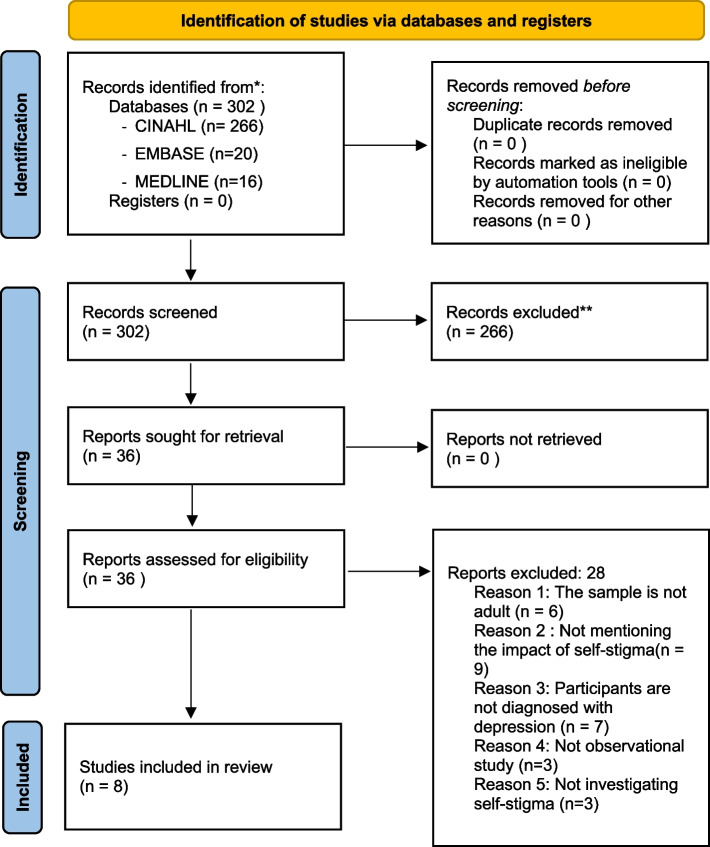
Search results

The search process resulted in the identification of 302 studies. The titles and abstracts were scanned for the total identified studies. Thirty-six studies appeared suitable for this review; however, after comparing them with the inclusion criteria for the current review, eight studies were included and twenty-eight were excluded for not fulfilling the inclusion criteria. The search process was done with the library team’s assistance at the University of Nottingham. The research characteristics of the included studies are illustrated in Table 2. The sequence of the search results’ process is illustrated through PRISMA 2020 flow diagram in Fig. 1.

**Table 2:** Research characteristics of the included studies

	**1**					**2**			**3**			**4**		
**Title**	Self-stigma and quality of life in patients with depressive disorder					Comparison of self-stigma and quality of life in depressive disorder and schizophrenia			Self-stigma formation process among younger and older Israeli Arabs diagnosed with depression			Self-stigma in patients with major depressive disorder: An exploratory study from India		
**Author and year of publication**	(Holubova et al., 2016b)					(Holubova et al., 2016a)			(Abo-Rass, Werner and Shinan-Altman, 2021)			(Patra et al., 2022)		
**Country**	Europe (Czech)					Europe (Czech)			Middle East (Israel)			Asia (India)		
**Total number of participants**	**81** patients with depression		**43** healthy controls participants			**80** patients with depression			**160** patients with depression			**50** patients with depression		
**Gender**	**M**	**F**	**M**		**F**	**M**	**F**		**M**	**F**		**M**	**F**	
	19	62	16		27	21	59		47	113		29	21	
**Age**	Adult aged 18 years and above					Adult age 18 to 60 years old			Adult age 18 to 75 years old			Adult age 18 to 65 years old		
**Methods**	Cross-sectional					Cross-sectional			Cross-sectional			Cross-sectional		
**Methods of data collection**	Questionnaire					Questionnaire			Interview			Interview		
**Exposure measure**	ISMI					ISMI			SSMIS			DISC		
**Outcome measure**	Q-LES-Q					Q-LES-Q CGI			RSES			WHODAS 2.0		
**Study Focus**	Depression only					Depression and schizophrenia			Depression only			Depression only		
**Data Analysis**	Regression analysis					Regression analysis			SPSS (version 25)			Regression analysis		
**Study conclusion**	Self‐stigma has a negative impact on **QoL**					Self‐stigma has a negative impact on **QoL**. The level of self-stigma in schizophrenia is **higher** than depression			Depression self-stigma has negative influence on **self-esteem**			Depression self-stigma has negative influence on **self-worthiness**		
	**5**					**6**			**7**			**8**		
**Title**	Quality of life among patients with depression: Impact of self-stigma					Self-stigma by people diagnosed with schizophrenia, depression and anxiety			Self-stigma in depressive patients: Association of cognitive schemata, depression, and self-esteem			Self-stigma in BPD comparison with schizophrenia, depressive disorder, and anxiety		
**Author and year of publication**	(Garg and Kaur, 2020)					(Hasan and Musleh, 2018)			(Shimotsu and Horikawa, 2016)			(Grambal et al., 2016)		
**Country**	Asia (India)					Middle east (Jordan)			Asia (Japan)			Europe (Czech)		
**Total number of participants**	**150** patients with depression					**119** patients with depression			**110** patients with depression			**33** patients with depression		
**Gender**	**M**			**F**		**M**		**F**	**M**		**F**	**M**		**F**
	64			86		54		65	54		56	18		15
**Age**	Adult aged 18 years and above					Adult age 18 to 52 years old			Adult age, mean age = 45.65 years			Adult age 18 to 60 years old		
**Methods**	Cross-sectional					Cross-sectional			Cross-sectional			Cross-sectional		
**Methods of data collection**	Interview					Questionnaire + Interview			Questionnaire			Questionnaire		
**Exposure measure**	The Hindi self‐stigma scale					Likert scale questionnaire			DDS			ISMI		
**Outcome measure**	WHO QoL Hindi version								RSES			CGI		
**Study Focus**	Depression only					Depression, Schizophrenia and Anxiety			Depression only			BPD, schizophrenia, depression and anxiety		
**Data analysis**	IBM SPSS (version 22.0)					SPSS (version 23)			- Correlation analysis - SEM			- GraphPad prism (version 5) - SPSS (version 24)		
**Study conclusion**	Self‐stigma has a negative impact on **QoL**					Depression self-stigma has negatively affected patients by feeling of **self-blame** The level of self-stigma in schizophrenia is **higher** than depression and anxiety			Depression and self-stigma have negatively influence on **self-esteem**			Impact of self-stigma on **social relations.** The level of self-stigma in patients with depression was the **second highest** level after BPD

The results of this review showed that there is a significant impact of depression self-stigma on QoL with a variant impact on the aspects of social relations, school, and study. Low self-esteem was reported as an impact of depression self-stigma; however, a single study indicates that low self-esteem is a symptom of depression, which could be due to depression itself or self-stigma. Self-blame and worthlessness were shown as an effect of self-stigma as the patients reported that they prevented themselves from taking an action in at least one life domain due to their mental illness condition. Concerning the comparison of self-stigma levels among patients with mental illness, most studies showed that schizophrenia was higher in experiencing self-stigma of MIS. A summary of the results was represented in Table 3.

**Table 3:** Summary of the results

**Category**	**Results**	**Sstudies reported the results**	
The impact of self-stigma on the QoL	Depression self-stigma has general negative impact on the QoL	Garg and Kaur, 2020 Holubova et al. 2016a Holubova et al. 2016b	
	Depression self-stigma has remarkable negative impact on social life and social relations	Grambal et al. 2016 Holubova et al. 2016a Patra et al. 2022	
	Depression self-stigma has insignificant negative impact on social life and social relations	Garg and Kaur, 2020 Holubova et al. 2016b	
	Depression self-stigma has less negative impact on academic performance and educational level for patients with depression	Holubova et al. 2016a Holubova et al. 2016b Patra et al. 2022	
	Self-stigma correlated insignificantly with stigma resistance and positive aspects of self-stigma	Garg and Kaur, 2020 Holubova et al. 2016b	
	Patients with depression self-stigma reported negative stereotypes due to their mental illness, which led to poor functioning in their lives	Hasan and Musleh, 2018 Holubova et al. 2016a Holubova et al. 2016b	
	Patients with depression tend to hide their mental illness due to their feelings of shame and self-stigmatization	Garg and Kaur, 2020 Patra et al. 2022	
The impact of self-stigma on the self-esteem	Lower levels of self-esteem were significantly associated with depression self-stigma		Abo-Rass et al. 2021 Garg and Kaur, 2020
	Low self-esteem is a symptom of depression and could be due to depression itself or to self-stigma		Shimotsu and Horikawa, 2016
The impact of self-stigma on the self- worthiness	76% of participants with depression reported anticipated discrimination and stopped themselves in at least one life domain		Patra et al. 2022
	32% of patients with depression anticipated discrimination, and they did not apply for jobs		
	Patients with depression reported self-blame for their mental condition		Hasan and Musleh, 2018
Comparing the level of depression self- stigma with self- stigma levels of other mental illnesses	Patients with schizophrenia stigmatized themselves more than patients with depression		Hasan and Musleh, 2018 Holubova et al. 2016a
	Patients with BPD stigmatized themselves more than psychiatric patients with schizophrenia, depression and anxiety		Grambal et al. 2016
	Depression recorded the highest rate in stigma resistance among all the diagnostic groups (BPD, schizophrenia and anxiety)		
	Patients blaming their mental illness was higher in patients with depression than in patients with schizophrenia and depression		Hasan and Musleh, 2018

### Findings of the included studies

This section represents and integrates the findings of the included studies that investigated the effect of self-stigma on the life aspects of patients with depression. The following domains were presented in this section: quality of life, self-esteem and self-worthiness. In addition, a comparison of the level of self- stigma of depression with the self-stigma of other mental illnesses was discussed.

#### Quality of Life (QoL)

The following studies showed the impact of self-stigma of depression on the QoL.

#### Holubova et al. 2016b [29]

In this study, the overall outcome of ISMI scale showed high significant correlation with all the domains of Q-LES-Q except of the school/study domain. In addition, two subscales from the ISMI scale (stereotype endorsement and stigma resistance) were not significantly correlated with the majority of the Q-LES-Q domains (see Table 4). The stereotype endorsement subscale was intended to measure the participants admitting to the common stereotypes of a patient with mental and psychological disorders, such as “psychiatric patients can not contribute and associate with the community because they are mentally ill” [28]. Stereotype endorsement was significantly correlated with the all the domains of Q-LES-Q except of the physical health, school/study, leisure, and social activities domains. The stigma resistance subscale was placed to determine the patient’s experience of resisting self-stigma of mental illness, such as “despite having a mental condition, I can live a happy and fulfilling life” [28]. This subscale correlated with all the domains of Q-LES-Q, but not with physical health, leisure time, social activities, and general domain.

**Table 4:** Relationship between self-stigma and QoL

	**Self-stigma Subscale**	**QoL Domains**									
		Physical health	Feelings		Work	Household	School/study	Leisure	Social activities	General	Sum of Q-les-Q
**Holubova et al. 2016b**	Overall score of ISMI	-**0.38** ***	-**0.47** ***		-**0.35** **	-**0.30** **	-0.10	-**0.26** *	-**0.33** **	-**0.42** ***	-**0.48** ***
	Alienation	-**0.41** ***	-**0.42** ***		-**0.25** *	-**0.33** **	-0.17	-**0.32** **	-**0.35** **	-**0.46** ***	-**0.46** ***
	Stereotype endorsement	-0.21	- **0.33** **		-**0.23** *	-**0.26** *	-0.002	-0.08	-0.17	-**0.24** *	-**0.28** *
	Perceived discrimination	-**0.27** *	-**0.24** *		-**0.37** ***	-0.16	-0.07	-0.13	-**0.24** *	-**0.24** *	-**0.32** **
	Social withdrawal	-**0.38** ***	-**0.45** ***		-**0.27** *	-**0.31** **	0.04	-**0.27** *	-**0.43** ***	-**0.38** ***	-**0.43** ***
	Stigma resistance	-0.21	-**0.28** *		-**0.28** *	-**0.23** *	-**0.34** *	-0.11	-0.04	-0.20	-**0.30** **
**Holubova et al. 2016a**	Overall score of ISMI	− 0.44***	− 0.56***		− 0.36***	− 0.41***	− 0.11	− 0.30***	− 0.44***	− 0.49***	− 0.56***
	Alienation	− 0.39***	− 0.50***		− 0.28***	− 0.34***	− 0.11	− 0.30***	− 0.37***	− 0.47***	− 0.49***
	Stereotype endorsement	− 0.37***	− 0.48***		− 0.29***	− 0.35***	− 0.06	− 0.23**	− 0.31***	− 0.37***	− 0.45***
	Perceived discrimination	− 0.31***	− 0.37***		− 0.31***	− 0.28***	− 0.09	− 0.20*	− 0.32***	− 0.35***	− 0.41***
	Social withdrawal	− 044***	− 0.50***		− 0.33***	− 0.35***	− 0.08	− 0.29***	− 0.50***	− 0.43***	− 0.52***
	Stigma resistance	− 0.29***	− 0.37***		− 0.18*	− 0.30***	− 0.19*	− 0.20*	− 0.18*	− 0.30***	− 0.35***

*P,0.05

**P,0.01

***P,0.001

#### Holubova et al. 2016a [15]

The overall result corresponded to the previous study of Holubova et al. (2016b) [29]. All the subscales of self-stigma were significantly negatively correlated with all the QoL domains except for the domain of school/study (see Table 4). This study indicates that patients who self-stigmatize tend to label themselves as “inferior, incompetent at fulfilling their needs and roles, limited in their skills and general life functioning and unable to succeed in life” (Holubova et al. 2016a, p. 3027) [15]. The patients’ self-evaluation of their mental status appears to have an impact on their perception of ability to function in many aspects of life.

#### Garg and Kaur, 2020 [30]

In this study, the total result of self-stigma was significantly negatively correlated with three domains of WHO QoL (satisfaction with psychological health, physical health and environment) (see Table 5). The correlation with satisfaction with social relations, however, was also negative but not significant. The majority of QoL subscales negatively correlated in a highly significant manner with the discrimination subscale. The discrimination subscale of the self‐stigma scale was significantly negatively correlated with all the domains of the WHO QoL, except one domain about the satisfaction with social relations. In addition, the subscales of stigma of disclosure and positive aspects of stigma were negative, although they did not reach statistical significance. The other subscale is assessing the positive aspects of stigma, such as, “my mental health problems have made me more accepting of other people” [31]. Garg and Kaur (2020) highlight that SSD has a negative impact on QoL [30]. “Stigma causes a significant reduction in hope, self‐esteem, socio‐occupational functioning, life opportunities resulting in shame, guilt, social isolation, and segregation” (Garg and Kaur, 2020, p. 128) [30].

**Table 5:** Relationship between self-stigma and QoL

**Garg and Kaur, 2020**	**Self-stigma Subscale**	**QoL Domains**					
		overall QoL	overall health	satisfaction with physical capacity	satisfaction with psychological health	satisfaction with social relations	satisfaction with environment
	Discrimination	−0.429**	−0.378**	−0.296**	−0.413**	−0.016	−0.317**
	Disclosure	−0.049	−0.019	−0.023	−0.137	−0.291	−0.058
	Positive aspects	−0.146	−0.284	−0.518	−0.805	−0.912	−0.538
	Total stigma scale score	−0.902**	−0.473**	−0.918**	−0.825*	−0.314	−0.178*

*P<0.5‐Significant

**P<0.05‐Highly Significant

#### Grambal et al. 2016 [32]

The social relationships are part of the QoL. This study showed an increase in self-stigma between divorced and single participants. Grambal et al. (2016) assumed that more self-stigmatised patients have problems starting and maintaining a close relationship. Consequently, there is a positive correlation between self-stigma and difficulties in establishing social relationships.

Hence, all the included studies showed an impact of self-stigma on the QoL. Holubova et al. (2016a) and Holubova et al. (2016b) agreed that self-stigma is significantly negatively correlated with QoL except in the school and study domain [15, 29]. Garg and Kaur (2020) indicate the significant influence of self-stigma on the QoL except for the insignificant impact on the social relationships [30]. However, Grambal et al. (2016) place emphasis on the impact and relation of self-stigma on social relationships. The studies’ results concerning the impact of self-stigma on QoL are displayed in Table 4.

### Self-esteem

The following studies showed an impact of depression self-stigma on self-esteem.

#### Abo-Rass et al. 2021 [33]

This study confirmed lower levels of self-esteem were significantly associated with self-stigma of depression. Specifically, SSMIS subscales (stereotype agreement and self-concurrence) were significantly negatively correlated with self-esteem. Stereotype agreement occurs when psychiatric patients support and agree with the common public stereotypes (e.g., mentally ill patients are weak). Self-concurrence is when the psychiatric patients apply and admit the cultural beliefs and views to him or herself (e.g., I am feeble because I have a psychiatric disorder) [34]. Moreover, in this study self- concurrence had the greatest relationship with low self-esteem.

#### Shimotsu and Horikawa, 2016 [35]

This study indicates that low self-esteem is one of the symptoms of depression. Therefore, depression and self-stigma have similar effect on self-esteem. This means depression in isolation could lead to low self- esteem. Additionally, self-stigma might lead to low self-esteem as well.

### Self-worthiness

Two of the included studies displayed the impression of self-stigma of depression on self-worthiness and self-blame.

#### Patra et al. 2022 [36]

This study showed that self-stigma of depression works as an obstacle to obtain professional career, social interaction and functional recovery that resulted in the following findings. 76% of participants with depression informed anticipated discrimination and stopped themselves in at least one life domain (such as making friends) due to their expectation of discrimination from others. 70% of the participants had hidden their mental illness from others. 54% had prevented themselves from having a close personal relationship. 32% anticipated discrimination, and they did not apply for jobs. 10% anticipated discrimination, and they did not complete their education. The previous findings confirmed that patients had self-worthlessness due to self-stigma of depression.

#### Hasan and Musleh, 2018 [27]

In this study, participants reported a higher response towards negative stereotypes, followed by patients’ self-blame for their mental condition, and a lower response for the factor ‘inability to recover’, which indicates that patients believe they will not improve.

### Comparison

This section will review the results of the included studies that concerned the comparison of self-stigma between depression and other mental illnesses. The following studies already discussed the matter of comparing self-stigma among mental illnesses, in addition to the main research aim; investigating the self-stigma impact.

#### Holubova et al. 2016a [15]

The overall ISMI score showed that participants with schizophrenia experienced high level of self-stigma more than participants with depression, specifically in the subscales for stereotype endorsement and perceived discrimination. The differences in alienation and social withdrawal subscales between schizophrenia and depression were markedly close, but schizophrenia was higher than depression.

#### Grambal et al. 2016 [32]

In this research, the participants with BPD showed the highest the level of self-stigma of all compared psychiatric disorders (schizophrenia, depression and anxiety). All subscales of ISMI showed the highest rate among participants with BPD except for one subscale (stigma resistance). Depression had the highest rate in stigma resistance among all the diagnostic groups.

#### Hasan and Musleh, 2018 [27]

This study aimed to compare depression, schizophrenia and anxiety. Hasan and Musleh (2018) indicated that the first subscale factor ‘negative stereotypes’ was considerably higher in schizophrenia than in depression and anxiety. Furthermore, negative stereotypes were significantly higher in depression than anxiety. Regarding the second subscale factor ‘patient blame’, depression was higher than both diagnostic groups (schizophrenia and anxiety). In the third subscale factor ‘inability to recover’, schizophrenia scored significantly higher than depression and anxiety. Hence, two of the included studies showed that self-stigma of schizophrenia was higher than self-stigma of depression and anxiety disorder. One study, however, showed that the level of self-stigma in BPD was higher than in schizophrenia, depression and anxiety disorder.

## Discussion

All the included studies assessed and addressed the impact of self-stigma on adult patients with depression. The impact was found to lower three aspects of the participants’ life and personality: QoL, self-worthiness and self-esteem. The QoL and self-worthiness were negatively influenced by the self-stigma of depression. Self-esteem varied slightly; some studies claimed that self- stigma led to low self-esteem, and a single study stated that depression in isolation could lead to lower self-esteem without self-stigma. Regarding the self-stigma comparison, most of the included studies found that schizophrenia patients showed higher levels of self-stigmatization. However, an individual study indicated that patients with BPD have higher levels of self-stigma, followed by schizophrenia, then, depression. Nevertheless, depressive disorder reported higher levels of patients’ self-blame and stigma resistance. A previous study found that self-stigma was associated with low QoL among patients with depressive disorders [37]. In general, internalized stigma led to lower QoL regardless of the type of mental illness. Further, the fact of being a person who sought psychiatric help could develop a negative self-image. As deduced from this review, the hospitalized patients reported higher levels of internalized stigma, which accordingly exposed them to the consequences of self-stigma, such as poor QoL [29]. In regard to social life, this review explored patients’ challenges while attempting to establish a social relationship. A study examined the impact of social interaction (SI) on 104 adult patients with severe mental illnesses [38]. This study found that negative SI significantly led to lower QoL, while supportive SI was related to higher QoL [38]. Further, they found that perceived stigma fairly liaised between negative SI and poor QoL [38]. The difference between negative and supportive SI on the patients’ QoL justifies why this review reported variant results about the impact of SSD on social life. Perhaps some participants received supportive SI and public acceptance, unlike others. Moreover, patients who received negative SI might prevent themselves from social interaction or building social relationships since they predict discrimination based on a previous experience. Anticipating discrimination is reported in this review as a behaviour observed by patients with depression who are identified to have SSD. This behaviour will encourage patients to hide their mental illness to prevent discrimination. Concealing mental illness by psychiatric patients is often appears with self-stigma of mental illness and it is the opposite of ‘stigma disclosure’. This review showed that hiding stigma is common in depressive disorders. Patients with depression tend to hide their mental illness due to their feelings of shame and self-stigmatization, which creates challenges and burdens for the patients while they try to cope with their lives and hide their mental disabilities. Hence, the elimination of mental illness stigma could be obtained by revealing the mental illness history of the affected person [18]. In regard to the self-esteem, a study reported high levels of self- esteem in self-stigmatized patients who received peer support [39]. Furthermore, family and peer support played a significant role in reducing self-stigma. The current review showed high levels of self- stigmatization among patients with depression who lived with their unsupportive parents in Asian and Middle Eastern countries. A recent systematic review aimed to investigate the frequency of mental illness self-stigma in different cultural and geographic areas [40]. The study found that the highest frequency was in South-East Asia (39.7%) and the Middle East (39%). Mental health problems are often neglected and hidden in Asian culture because admitting mental illness is usually associated with shame, stigmatization and lack of family support [41]. Furthermore, McGuire and Pace (2018) studied the impact of self-stigma of depression between Christians and non-Christian participants diagnosed with depression [42]. The study showed an increased level of self-stigma in the Christian group, which reveals that religious and cultural beliefs impacted the self- stigma of depression. Regarding self-worth, Corrigan, Larson and Rusch (2009) identified the “Why Try” effect of self-stigma [43]. This effect occurred when people with psychiatric disorders considered themselves unworthy or unqualified to achieve life goals due to their application of public mental illness stereotypes to themselves [44]. Corrigan et al. (2009) [43] showed that negative self-worth was observed highly in patients with depression and linked with offensive stereotypes to themselves [34]. An example of the patient’s thought is “someone like me is just not worth to be successful in life” [43]. Correspondingly, the findings of the current review reported reduction in self-worth due to SSD. The forms of self-worthlessness include the patients’ refusal to get a job and to blame themselves for their mental illness. In this review, the level of self-stigma was altered among mental illnesses, but most of the included studies reported a high level of internalized stigma in schizophrenia, except one study reported high level of stigma on BPD, which could be due to the disorder’s symptoms as it is identified by disturbance of self-image, thoughts, and mood. This increased self-stigmatization in schizophrenia could be due to the evident influence of the public stigma on patients with psychotic disorders. Moreover, the public considered psychotic patients more dangerous and aggressive [45]. Moreover, schizophrenia is characterized by hallucinations and delusions, which are obvious symptoms that easily produce public stigma, unlike nonpsychotic [46]. Therefore, the effect of public stigma will extend to disturbed self-perception, which will result in high levels of internalized stigma. Some published studies, however, reported that nonpsychotic disorders had higher levels of self-stigma compared to psychotic disorders. A tested and confirmed hypothesis indicate that patients with nonpsychotic disorders such as depression were aware of the negative public stereotypes, making them experience self-stigma more intensely than psychotic patients [47]. From the same perspective, in this review self-stigma resistance was reported as high in depression. Also, a study showed that greater stigma resistance was found among patients with depression [48]. Stigma resistance in mental health is defined as the ability to remain unresponsive to mental illness stigma [28]. The resistance was associated with reduced self-stigma, increased self-esteem and improved QoL [49]. The presence of self-stigma resistance among patients with depression could be due to the patients’ awareness of public stigma since patients are aware they will be able to cope with internalised stigma. Some of the included studies, however, reported low stigma resistance, which could be interpreted as a response of depression symptoms such as feelings of worthlessness, anhedonia and irritable mood. Self-blaming is usually linked to depression. A study showed that more than 80% of patients with depression reported self-blaming for failing to achieve life duties such as losing jobs or social relations [50]. Thus, these reasons for self-blaming indicated poor QoL, which is mentioned earlier in this review as an impact of self-stigma. Clearly, self-stigma leads indirectly to self-blaming, and depression demonstrated a higher rate of self-blaming due to internalized stigma. Generalization of the research results is critical when applying the findings to population, settings, and times other than those in the original study. A research evidence can only be applied outside the contexts studied when the settings and population are similar to the original study, otherwise there would be no evidence-based practice [51]. Regarding this study, the culture was an obstacle to generalizing the study findings. The Asian studies in this review reported an impact of self- stigma on self-esteem and self-worth. In contrast, none of the European studies mentioned the effects of self-stigma on self-esteem or self-worth. Consequently, the self-stigma was affected by the culture, traditions and beliefs of the patients’ societies. For example, Asians tend to live with their families, making psychiatric patients more exposed to public stigma by their families, leading to self-esteem disturbance. In addition, three of the included studies in this review have confirmed cultural differences encountered while investigating the impact of self-stigma among patients with depression. Furthermore, A study confirmed cross-cultural differences in the relationship between mental illness self-stigma and other concepts, such as masculine sex norms and negative ideas toward seeking mental help [52]. Consequently, due to cultural differences, the findings of this review could not be globally generalized. Nonetheless, it could be locally generalized based on each cultural type and geographical site.

### Strengths and limitations

All the included studies that contributed to the outcomes of this review were observational (cross-sectional) studies. Cross-sectional studies are best used in health research to assess and investigate the exposure and outcome of a particular health issue [53]. Cross-sectional studies, however, are prone to some biases [54]. For example, the included studies of this review measured the self-stigma of mentally ill patients at one point during the interview or questionnaire, regardless of the patient’s compliance to medications, which were found to influence the onset and severity of self-stigma. According to this review, patients who adhered to medications experienced less self-stigma and more stigma resistance.

The main limitation of this review is that the issue of self-stigma of depression was not sufficiently discussed and covered in previous research. In contrast, the researchers broadly discussed the impact of self-stigma on patients with schizophrenia and bipolar disorder (BD). This attention could be due to increasing public stigma, which leads to increased internalized stigma for patients with schizophrenia and BD. Therefore, the researcher will be encouraged to study more about the self-stigma of schizophrenia or BD. Furthermore, four of the included studies stated that their study is the first of its type acknowledging the impact of self-stigma on patients with depression [27, 30, 32, 36]. This explains the limited number of included studies in this review. A strong point of this research is that it included recent studies despite the limited number of published papers on the topic. Thus, the need for the current study was essential and required. All the included studies comprised a sample from both genders. The ratio number of males and females was not close in most studies. Three studies reported insignificant differences between the genders. The remaining studies did not mention the differences they found. That could affect the reliability of the study. A study indicated that females with mental illnesses tend to self- stigmatize themselves more than males [55]. Additional limitations arise from the omission of techniques aimed at minimizing errors of data extraction, which has not been done due to the time constraints for this review. Another limitation pertains to the restricted scope of searching within identified databases, disregarding the inclusion of gray literature that has not been explored. Finally, the inclusion criteria of the study settings were broad, which was considered a limitation since it found that the hospitalized patients stigmatized themselves more than the psychiatric patients who visited the outpatient clinics.

## Conclusion

The current review satisfactorily answered the research question and met the objectives. The review explored the impact of SSD. The impact was demonstrated from the QoL to the profound intrapersonal effect on self-esteem and self-worthiness. The QoL was negatively affected and reflected on negative stereotypes by the patients themselves, which led to poor functioning in life. In addition, due to self- stigmatization, patients with depression tend to hide their mental illness, which is associated indirectly with decreasing the QoL. Moreover, self-stigma has a significantly negative impact on social life.

However, some studies have proven that it insignificantly negatively affects. Furthermore, SSD had a slightly negative effect on academic performance. Intrapersonal impact depicted lower levels of self-esteem and self-worthiness associated with SSD. This study has proven that the lower self-esteem level is hard to decide as an impact of SSD since it is a symptom of depression. Self-worthiness affected the patients by stopping them in at least one life domain. They anticipated discrimination and prevented themselves from taking decisions and getting better opportunities. Moreover, patients with depression reported self-blame for their mental condition. Studying the impact from a global perspective allowed the author to know the majority of the effects of SSD and understand the cultural differences affecting it. The Asian and Middle Eastern cultures showed increasing rates and severity of self-stigma of depression. This study emphasized that public and perceived stigma are associated with the formation of self-stigma. Further, the published studies in this area were heterogeneous regarding the self-stigma scales used, so generating systematic reviews and meta-analysis is recommended based on a unified self-stigma scale used in all of the included studies since this will provide accurate and reliable findings.

**Fig. 2:**
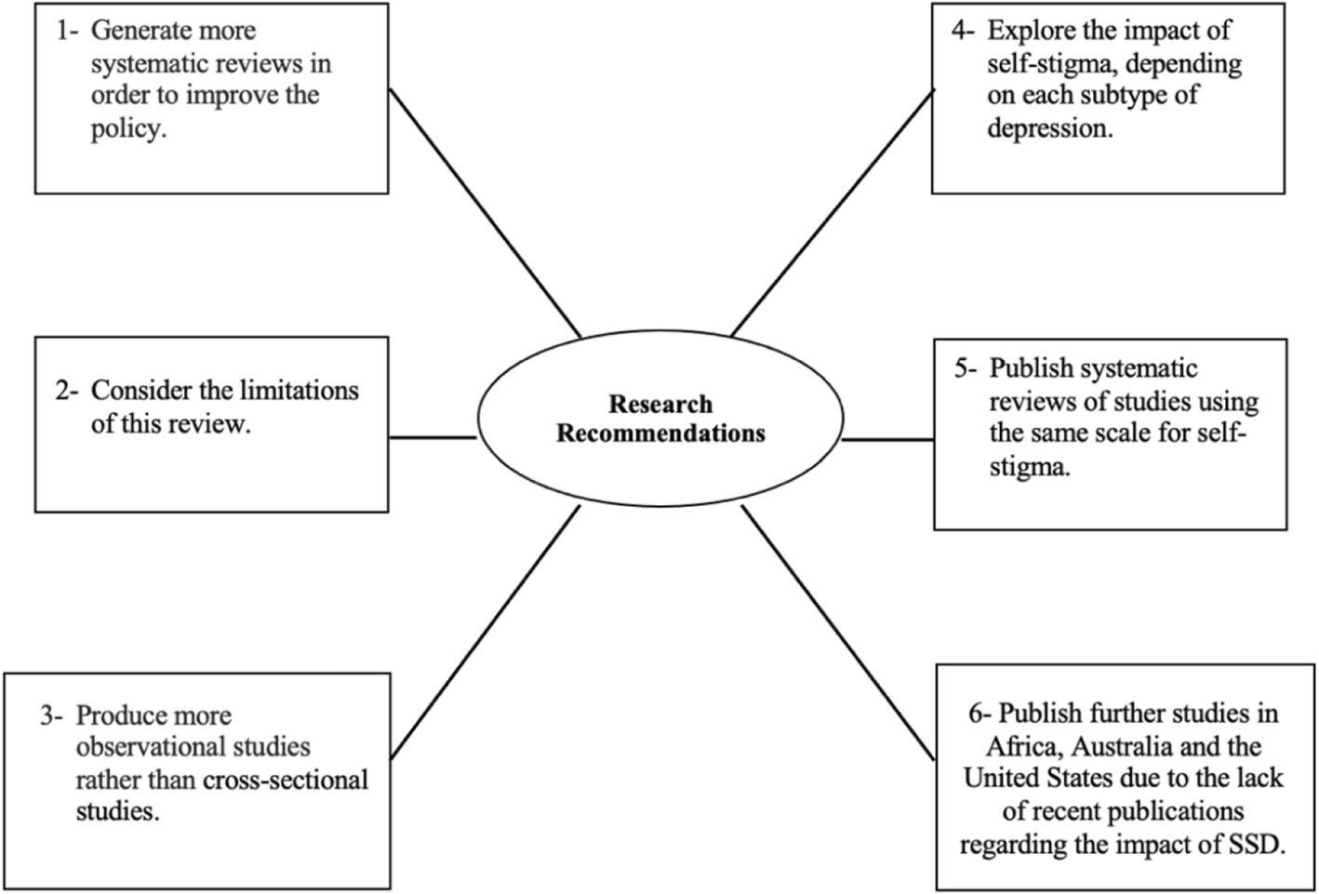
The research recommendations

**Fig. 3:**
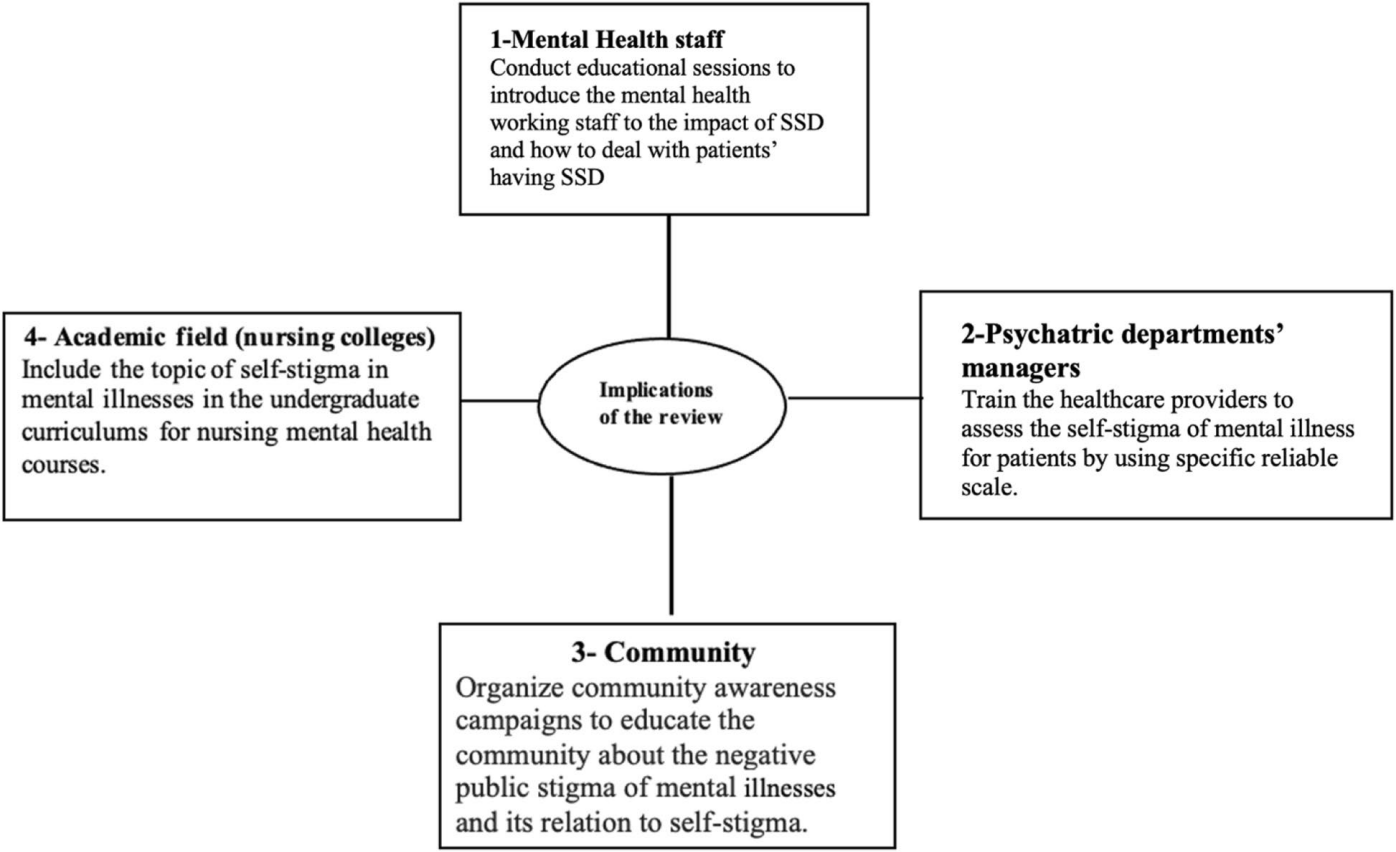
The clinical implications

The research recommendations and clinical implications of this review were summarized and presented in Figs. 2 and 3.

### Supplementary Information

Supplementary Material 1.

Supplementary Material 2.

Supplementary Material 3.
Supplementary Material 4.

## Data Availability

Data is provided within the manuscript or supplementary information files and The datasets analysed during the current study are available from the corresponding author on reasonable request.

## Abbreviations

BD: Bipolar Disorder
BPD: Borderline Personality Disorder
CINAHL: Cumulative Index to Nursing and Allied Health Literature
EMBASE: Excerpta Medica Database
JBI: Joanna Briggs Institute
MEDLINE: Medical Literature Analysis and Retrieval System Online
MIS: Mental Illness Stigma
QoL: Quality of Life
SI: Social Interaction
SSD: Self-stigma of Depression
WHO: World Health Organization
