# Supplementary material for: The general impact of self-stigma of mental illness on adult patients with depressive disorders: a systematic review
Source: BMC Nurs. 2024 Jun 25;23:432. doi: 10.1186/s12912-024-02047-z (PMC11200989; doi:10.1186/s12912-024-02047-z)
Supplement: Supplementary file 1 — Supplementary Material 1. [file 12912_2024_2047_MOESM1_ESM.docx]

# Appendix 1: The search strategy and keywords for the used databases.

- The search keywords

| **Key concept** | **Synonyms and related terms** |
| --- | --- |
| Depression | depression OR depressive OR depressi* OR depressi* disorders |
| Self-stigma | self-stigma OR depression self-stigma OR internalized stigma OR  depression internalized stigma |
| Adult patients | Adults OR adult patients OR adult psychiatric patients OR adult  population OR adult participants |
| Impact | influence OR impact OR effect |

- Keywords application on the databases.

CINAHL Plus with full text.

| **Search**  **No.** | **Search terms** | **Results** |
| --- | --- | --- |
| 1 | depression | (7650) |
| 2 | depressive | (1935) |
| 3 | depressi* | (7902) |
| 4 | depressi* disorders | (900) |
| 5 | 1 OR 2 OR 3 OR 4 | (7902) |
| 6 | Limit 5 to yr.= (2016-2022), Limit 5 to English language | (1601) |
| 7 | self-stigma | (31) |
| 8 | depression self-stigma | (0) |
| 9 | internalized stigma | (7) |
| 10 | depression internalized stigma | (0) |
| 11 | 7 OR 9 | (31) |
| 12 | Limit 11 to yr.= (2016-2022), Limit 11 to English language | (19) |
| 13 | Adults | (101,876) |
| 14 | adult patients | (188) |
| 15 | adult psychiatric patients | (14) |

| 16 | adult population | (1437) |
| --- | --- | --- |
| 17 | adult participants | (1099) |
| 18 | 13 OR 14 OR 15 OR 16 OR 17 | (101,876) |
| 19 | Limit 18 to yr.= (2016-2022), Limit 18 to English language | (14,744) |
| 20 | influence | (83,606) |
| 21 | impact | (94.263) |
| 22 | effect | (133,450) |
| 23 | 20 OR 21 OR 22 | (263,241) |
| 24 | Limit 23 to yr.= (2016-2022), Limit 23 to English language | (63,019) |
| 25 | (5) and (11) and (18) and (23) | (206,683) |
| 26 | Limit 25 to yr.= (2016-2022), Limit 25 to English language | (266) |

EMBASE

| **Search**  **No.** | **Search terms** | **Results** |
| --- | --- | --- |
| 1 | Depression/ | (434,678) |
| 2 | Depressive.mp. | (194,142) |
| 3 | depressi* .mp. | (818,116) |
| 4 | depressi* disorders. mp. | (16,653) |
| 5 | 1 OR 2 OR 3 OR 4 | (818,116) |
| 6 | Limit 5 to yr.= (2016-2022), Limit 5 to English language, Limit  5 to full text | (35,834) |
| 7 | self-stigma.mp. | (1337) |
| 8 | depression self-stigma.mp. | (7) |
| 9 | internalized stigma.mp. | (1142) |
| 10 | depression internalized stigma | (6) |
| 11 | 7 OR 8 OR 9 OR 10 | (2259) |
| 12 | Limit 11 to yr.= (2016-2022), Limit 11 to English language,  Limit 11 to full text | (175) |
| 13 | Adults/ | (8,528,329) |
| 14 | adult patients.mp. | (169,760) |
| 15 | adult psychiatric patients.mp. | (300) |

| 16 | adult population.mp. | (34283) |
| --- | --- | --- |
| 17 | adult participants. mp. | (6781) |
| 18 | 13 OR 14 OR 15 OR 16 OR 17 | (7,764,789) |
| 19 | Limit 18 to yr.= (2016-2022), Limit 18 to English language,  Limit 18 to full text | (529,175) |
| 20 | Influence.mp. | (1,401,107) |
| 21 | Impact.mp. | (1,789,390) |
| 22 | Effect.mp. | (6,096,488) |
| 23 | 20 OR 21 OR 22 | (8,405,618) |
| 24 | Limit 23 to yr.= (2016-2022), Limit 23 to English language,  Limit 23 to full text | (337,928) |
| 25 | (5) and (11) and (18) and (23) | (179) |
| 26 | Limit 25to yr.= (2016-2022), Limit 25to English language,  Limit 25 to full text | (20) |

MEDLINE

| **Search**  **No.** | **Search terms** | **Results** |
| --- | --- | --- |
| S1 | Depression or depressive or depressi* or depressi* disorders | (488.894) |
| S2 | depressive | (203,561) |
| S3 | depressi* | (534,026) |
| S4 | depressi* disorders | (158,005) |
| S5 | S1 OR S2 OR S3 OR S4 | (534,026) |
| S6 | Limit S5 to yr.= (2016-2022), Limit S5 to English language,  Limit S5 to age related, Limit S5 to all Adult population. | (73.622) |
| S7 | self-stigma | (1,098) |
| S8 | depression self-stigma | (52) |
| S9 | internalized stigma | (1,264) |
| S10 | depression internalized stigma | (92) |
| S11 | S7 OR S8 OR S9 OR S10 | (2177) |
| S12 | Limit S11 to yr.= (2016-2022), Limit S11 to English language,  Limit S11 to age related, Limit S11 to all Adult population. | (824) |

| S13 | Adults | (6,332,460) |
| --- | --- | --- |
| S14 | adult patients | (181,115) |
| S15 | adult psychiatric patients | (807) |
| S16 | adult population | (62,671) |
| S17 | adult participants | (22,646) |
| S18 | S13 OR S14 OR S15 OR S16 OR S17 | (6,332,460) |
| S19 | Limit S18 to yr.= (2016-2022), Limit S18 to English language,  Limit S18 to age related, Limit S18 to all Adult population. | (1,200,522) |
| S20 | influence | (1,348,792) |
| S21 | impact | (1,351,099) |
| S22 | effect | (9,208,856) |
| S23 | S20 OR S21 OR S22 | (10,702,6030 |
| S24 | Limit S23 to yr.= (2016-2022), Limit S23 to English language,  Limit S23 to age related, Limit S23 to all Adult population. | (707,739) |
| S25 | (S5) and (S11) and (S18) and (S23) | (212) |
| S26 | Limit S25 to yr.= (2016-2022), Limit S25 to English language, Limit S11 to age related, Limit S11 to all Adult population.  Limit S25 to full text. | (16) |

**Appendix 2 : Critical appraisal checklist for all the included studies.**

**Author**: Holubova, M., Prasko, J., Ociskova, M., Marackova, M., Grambal, A. and Slepecky, M. (Holubova et al., 2016a). **Year**: 2016

Overall appraisal: 6 Include: ✓ Exclude: - Seek further info: -

| JBI critical appraisal checklist for analytical cross-sectional studies | | | | | |
| --- | --- | --- | --- | --- | --- |
| **Items** | | **Yes**  **(1)** | **No**  **(0)** | **Unclear**  **(0.5)** | **Not**  **applicable** |
| 1- Were the criteria for inclusion in the sample clearly defined? | | ✓ |  |  |  |
| 2- | Were the study subjects and the setting described in detail? | ✓ |  |  |  |
| 3- | Was the exposure measured in a valid and reliable way? | ✓ |  |  |  |
| 4- Were objective, standard criteria used for measurement of the condition? | | ✓ |  |  |  |
| 5- | Were the outcomes measured in a valid and reliable way? | ✓ |  |  |  |
| 6- | Was appropriate statistical analysis used? | ✓ |  |  |  |
| **Total Score** | | **6/6** | | | |

Comments:

The study by Holubova et al., 2016b is included in the current review as it fulfilled all the inclusion criteria and addressed the subject of self-stigma and quality of life in patients with depressive disorder. The exposure and outcome were measured in reliable and valid methods (Internalized Stigma of Mental Illness scale (ISMI) and Quality of Life and Enjoyment Questioner (Q-LES-Q). The regression analysis was used for the statistical analysis. The research by Holubova et al. (2016b) answered the main question of the current review and proved that self‐stigma has a negative impact on QoL.

**Author**: Holubova, M., Prasko, J., Matousek, S., Latalova, K., Marackova, M., Vrbova, K., Grambal, A., Slepecky, M. and Zatkova, M. (Holubova et al., 2016a) **Year**: 2016

Overall appraisal: 6 Include: ✓ Exclude: - Seek further info: -

| JBI critical appraisal checklist for analytical cross-sectional studies | | | | | |
| --- | --- | --- | --- | --- | --- |
| **Items** | | **Yes**  **(1)** | **No**  **(0)** | **Unclear**  **(0.5)** | **Not**  **applicable** |
| 1- Were the criteria for inclusion in the sample clearly defined? | | ✓ |  |  |  |
| 2- | Were the study subjects and the setting described in detail? | ✓ |  |  |  |
| 3- | Was the exposure measured in a valid and reliable way? | ✓ |  |  |  |
| 4- Were objective, standard criteria used for measurement of the condition? | | ✓ |  |  |  |
| 5- | Were the outcomes measured in a valid and reliable way? | ✓ |  |  |  |
| 6- | Was appropriate statistical analysis used? | ✓ |  |  |  |
| **Total Score** | | **6/6** | | | |

Comments:

The study by Holubova et al., 2016a is included in the current review as it fulfilled all the inclusion criteria and addressed the subject of comparing of self-stigma and quality of life in depressive disorder and schizophrenia. The exposure and outcome were measured in reliable and valid methods (Internalized Stigma of Mental Illness scale (ISMI), Quality of Life and Enjoyment Questioner (Q-LES-Q) and Clinical Global Impression (CGI). The regression analysis was used for the statistical analysis. The research by Holubova et al. (2016a) answered the main question and objective of the current review and proved that Self‐stigma has a negative impact on QoL. Moreover, the research showed that the level of self-stigma in schizophrenia is higher than depression.

**Author**: Abo-Rass, Werner and Shinan-Altman. **Year**: 2021

Overall appraisal: 6 Include: ✓ Exclude: - Seek further info: -

| JBI critical appraisal checklist for analytical cross-sectional studies | | | | | |
| --- | --- | --- | --- | --- | --- |
| **Items** | | **Yes**  **(1)** | **No**  **(0)** | **Unclear**  **(0.5)** | **Not**  **applicable** |
| 1- Were the criteria for inclusion in the sample clearly defined? | | ✓ |  |  |  |
| 2- | Were the study subjects and the setting described in detail? | ✓ |  |  |  |
| 3- | Was the exposure measured in a valid and reliable way? | ✓ |  |  |  |
| 4- Were objective, standard criteria used for measurement of the condition? | | ✓ |  |  |  |
| 5- | Were the outcomes measured in a valid and reliable way? | ✓ |  |  |  |
| 6- | Was appropriate statistical analysis used? | ✓ |  |  |  |
| **Total Score** | | **6/6** | | | |

Comments:

The study by Abo-Rass et al. (2021) is included in the current review as it fulfilled all the inclusion criteria and addressed the subject of self-stigma formation process among younger and older Israeli Arabs diagnosed with depression. The exposure and outcome were measured in reliable and valid methods (self- Stigma of Mental Health Scale (SSMIS) and Rosenberg’s Self-Esteem Scale (RSES). The SPSS (version 25) was used for the statistical analysis. The research by Abo-Rass et al. (2021) answered the main question of the current review and showed that depression self-stigma has negative influence on self- esteem.

**Author**: Patra, B. N., Patil, V., Balhara, Y. and Khandelwal, S. K. **Year**: 2022 Overall appraisal: 6 Include: ✓ Exclude: - Seek further info: -

| JBI critical appraisal checklist for analytical cross-sectional studies | | | | | |
| --- | --- | --- | --- | --- | --- |
| **Items** | | **Yes**  **(1)** | **No**  **(0)** | **Unclear**  **(0.5)** | **Not**  **applicable** |
| 1- Were the criteria for inclusion in the sample clearly defined? | | ✓ |  |  |  |
| 2- | Were the study subjects and the setting described in detail? | ✓ |  |  |  |
| 3- | Was the exposure measured in a valid and reliable way? | ✓ |  |  |  |
| 4- Were objective, standard criteria used for measurement of the condition? | | ✓ |  |  |  |
| 5- | Were the outcomes measured in a valid and reliable way? | ✓ |  |  |  |
| 6- | Was appropriate statistical analysis used? | ✓ |  |  |  |
| **Total Score** | | **6/6** | | | |

Comments:

The study by Patra et al. (2022) is included in the current review as it fulfilled all the inclusion criteria and addressed the subject of self-stigma in patients with major depressive disorder in India. The exposure and outcome were measured in reliable and valid methods (Discrimination and Stigma Scale (DISC) and WHO Disability Assessment Schedule (WHODAS, 2.0). The regression analysis was used for the statistical analysis. The research by Patra et al. (2022) answered the main question of the current review and showed that depression self-stigma has negative influence on self-worthiness.

**Author**: Garg and Kaur. **Year**: 2020

Overall appraisal: 6 Include: ✓ Exclude: - Seek further info: -

| JBI critical appraisal checklist for analytical cross-sectional studies | | | | | |
| --- | --- | --- | --- | --- | --- |
| **Items** | | **Yes**  **(1)** | **No**  **(0)** | **Unclear**  **(0.5)** | **Not**  **applicable** |
| 1- Were the criteria for inclusion in the sample clearly defined? | | ✓ |  |  |  |
| 2- | Were the study subjects and the setting described in detail? | ✓ |  |  |  |
| 3- | Was the exposure measured in a valid and reliable way? | ✓ |  |  |  |
| 4- Were objective, standard criteria used for measurement of the condition? | | ✓ |  |  |  |
| 5- | Were the outcomes measured in a valid and reliable way? | ✓ |  |  |  |
| 6- | Was appropriate statistical analysis used? | ✓ |  |  |  |
| **Total Score** | | **6/6** | | | |

Comments:

The study by Garg and Kaur (2020) is included in the current review as it fulfilled all the inclusion criteria and addressed the subject of quality of life among patients with depression: impact of self-stigma. The exposure and outcome were measured in reliable and valid methods (the Hindi self‐stigma scale and WHO QoL Hindi version). The IBM SPSS (version 22.0) was used for the statistical analysis. The research by Garg and Kaur (2020) answered the main question of the current review and showed that depression self-stigma has negative impact on QoL

**Author**: Shimotsu and Horikawa. **Year**: 2016

Overall appraisal: 6 Include: ✓ Exclude: - Seek further info: -

| JBI critical appraisal checklist for analytical cross-sectional studies | | | | | |
| --- | --- | --- | --- | --- | --- |
| **Items** | | **Yes**  **(1)** | **No**  **(0)** | **Unclear**  **(0.5)** | **Not**  **applicable** |
| 1- Were the criteria for inclusion in the sample clearly defined? | | ✓ |  |  |  |
| 2- | Were the study subjects and the setting described in detail? | ✓ |  |  |  |
| 3- | Was the exposure measured in a valid and reliable way? | ✓ |  |  |  |
| 4- Were objective, standard criteria used for measurement of the condition? | | ✓ |  |  |  |
| 5- | Were the outcomes measured in a valid and reliable way? | ✓ |  |  |  |
| 6- | Was appropriate statistical analysis used? | ✓ |  |  |  |
| **Total Score** | | **6/6** | | | |

Comments:

The study by Shimotsu and Horikawa (2016) is included in the current review as it fulfilled all the inclusion criteria and addressed the subject of self-stigma in depressive patients: Association of cognitive schemata, depression, and self-esteem. The exposure and outcome were measured in reliable and valid methods (Devaluation-Discrimination Scale (DDS) and Rosenberg’s Self-Esteem Scale (RSES). The Correlation analysis and structural Equation Modelling were used for the statistical analysis. The research by Shimotsu and Horikawa (2016) answered the main question of the current review and showed that depression self- stigma has negative impact on the self-eseem.

**Author**: Grambal, A., Prasko, J., Kamaradova, D., Latalova, K., Holubova, M., Marackova, M., Ociskova, M. and Slepecky. **Year**: 2016

Overall appraisal: 6 Include: ✓ Exclude: - Seek further info: -

| JBI critical appraisal checklist for analytical cross-sectional studies | | | | | |
| --- | --- | --- | --- | --- | --- |
| **Items** | | **Yes**  **(1)** | **No**  **(0)** | **Unclear**  **(0.5)** | **Not**  **applicable** |
| 1- Were the criteria for inclusion in the sample clearly defined? | | ✓ |  |  |  |
| 2- | Were the study subjects and the setting described in detail? | ✓ |  |  |  |
| 3- | Was the exposure measured in a valid and reliable way? | ✓ |  |  |  |
| 4- Were objective, standard criteria used for measurement of the condition? | | ✓ |  |  |  |
| 5- | Were the outcomes measured in a valid and reliable way? | ✓ |  |  |  |
| 6- | Was appropriate statistical analysis used? | ✓ |  |  |  |
| **Total Score** | | **6/6** | | | |

Comments:

The study by Grambal et al. (2016) is included in the current review as it fulfilled all the inclusion criteria and addressed the subject of self-stigma in BPD comparison with schizophrenia, depressive disorder, and anxiety. The exposure and outcome were measured in reliable and valid methods (Internalized Stigma of Mental Illness scale (ISMI) and Clinical Global Impression (CGI). The GraphPad prism (version 5) and SPSS (version 24) were used for the statistical analysis. The research by Grambal et al. (2016) answered the main question and objective of the current review and showed that the self-stigma impacts negatively on the social relations. Moreover, the level of self-stigma in patients with depression was the second highest level after BPD.

**Author**: Hasan and Musleh. **Year**: 2018

| JBI critical appraisal checklist for analytical cross-sectional studies | | | | | |
| --- | --- | --- | --- | --- | --- |
| **Items** | | **Yes**  **(1)** | **No**  **(0)** | **Unclear**  **(0.5)** | **Not**  **applicable** |
| 1- Were the criteria for inclusion in the sample clearly defined? | | ✓ |  |  |  |
| 2- | Were the study subjects and the setting described in detail? | ✓ |  |  |  |
| 3- | Was the exposure measured in a valid and reliable way? |  | ✓ |  |  |
| 4- Were objective, standard criteria used for measurement of the condition? | | ✓ |  |  |  |
| 5- | Were the outcomes measured in a valid and reliable way? |  | ✓ |  |  |
| 6- | Was appropriate statistical analysis used? | ✓ |  |  |  |
| **Total Score** | | **4/6** | | | |

Overall Comments:

The study by Hasan and Musleh (2018) is included in the current review as it fulfilled all the inclusion criteria and addressed the subject of self-stigma by people diagnosed with schizophrenia, depression and anxiety. The exposure and outcome were not measured in clear and reliable methods, the authors used their own Likert scale questionnaire that has not tested or used before. The SPSS (version 23) was used for the statistical analysis. The research by Hasan and Musleh (2018) answered the main question and objective of the current review and showed that the depression self-stigma has negatively affected patients by feelings of self-blame. Moreover, the level of self-stigma in schizophrenia is higher than depression and anxiety.

appraisal: 4 Include: ✓ Exclude: - Seek further info: -

Appendix 3: Relationship between self-stigma and QoL

| **Holubova et al. 2016b** | | **Self-stigma Subscale** | | **QoL Domains** | | | | | | | | | | | | | | |
| --- | --- | --- | --- | --- | --- | --- | --- | --- | --- | --- | --- | --- | --- | --- | --- | --- | --- | --- |
|  |  |  |  | Physical health | | Feelings | | Work | | Household | School/study | | Leisure | Social  activities | | General | Sum of Q-les-Q | |
|  |  | Overall score of  ISMI | | -**0.38** *** | | -**0.47** *** | | -**0.35** ** | | -**0.30** ** | -0.10 | | -**0.26** * | -**0.33** ** | | -**0.42** *** | -**0.48** *** | |
|  |  | Alienation | | -**0.41** *** | | -**0.42** *** | | -**0.25** * | | -**0.33** ** | -0.17 | | -**0.32** ** | -**0.35** ** | | -**0.46** *** | -**0.46** *** | |
|  |  | Stereotype  endorsement | | -0.21 | | - **0.33** ** | | -**0.23** * | | -**0.26** * | -0.002 | | -0.08 | -0.17 | | -**0.24** * | -**0.28** * | |
|  |  | Perceived  discrimination | | -**0.27** * | | -**0.24** * | | -**0.37** *** | | -0.16 | -0.07 | | -0.13 | -**0.24** * | | -**0.24** * | -**0.32** ** | |
|  |  | Social withdrawal | | -**0.38** *** | | -**0.45** *** | | -**0.27** * | | -**0.31** ** | 0.04 | | -**0.27** * | -**0.43** *** | | -**0.38** *** | -**0.43** *** | |
|  |  | Stigma resistance | | -0.21 | | -**0.28** * | | -**0.28** * | | -**0.23** * | -**0.34** * | | -0.11 | -0.04 | | -0.20 | -**0.30** ** | |
| **Holubova et al. 2016a** | | Overall score of  ISMI | | −**0.44***** | | −**0.56***** | | −**0.36***** | | −**0.41***** | −0.11 | | −**0.30***** | −**0.44***** | | −**0.49***** | −**0.56***** | |
|  |  | Alienation | | −**0.39***** | | −**0.50***** | | −**0.28***** | | −**0.34***** | −0.11 | | −**0.30***** | −**0.37***** | | −**0.47***** | −**0.49***** | |
|  |  | Stereotype  endorsement | | −**0.37***** | | −**0.48***** | | −**0.29***** | | −**0.35***** | −0.06 | | −**0.23**** | −**0.31***** | | −**0.37***** | −**0.45***** | |
|  |  | Perceived  discrimination | | −**0.31***** | | −**0.37***** | | −**0.31***** | | −**0.28***** | −0.09 | | −**0.20*** | −**0.32***** | | −**0.35***** | −**0.41***** | |
|  |  | Social withdrawal | | −**044***** | | −**0.50***** | | −**0.33***** | | −**0.35***** | −0.08 | | −**0.29***** | −**0.50***** | | −**0.43***** | −**0.52***** | |
|  |  | Stigma resistance | | −0.29*** | | −0.37*** | | −0.18* | | −0.30*** | −0.19* | | −0.20* | −0.18* | | −0.30*** | −0.35*** | |
|  |  | *P,0.05; **P,0.01; ***P,0.001 | | | | | | | | | | | | | | | | |
| **Garg and Kaur, 2020** | | **Self-stigma Subscale** | | **QoL Domains** | | | | | | | | | | | | | | |
|  |  |  |  | overall QoL | | overall health | | satisfaction with physical capacity | | | satisfaction with psychological health | | | satisfaction with social relations | | | satisfaction with  environment | |
|  |  | Discrimination | | **−0.429**** | | **−0.378**** | | **−0.296**** | | | **−0.413**** | | | −0.016 | | | **−0.317**** | |
|  |  | Disclosure | | −0.049 | | −0.019 | | −0.023 | | | −0.137 | | | −0.291 | | | −0.058 | |
|  |  | Positive aspects | | −0.146 | | −0.284 | | −0.518 | | | −0.805 | | | −0.912 | | | −0.538 | |
|  |  | Total stigma scale  score | | **−0.902**** | | **−0.473**** | | **−0.918**** | | | **−0.825*** | | | −0.314 | | | **−0.178*** | |
|  |  | *P<0.5‐Significant; **P<0.05‐Highly Significant | | | | | | | | | | | | | | | | |

| **Study**  **No.** | **Author** | **Critical Appraisal Results**  (Yes: No: Unclear) | **Total score out of 6** |
| --- | --- | --- | --- |
| **1** | Holubova et al. 2016b | 6:0:0 | 6 |
| **2** | Holubova et al. 2016a | 6:0:0 | 6 |
| **3** | Abo-Rass, Werner and  Shinan-Altman, 2021 | 6:0:0 | 6 |
| **4** | Patra et al. 2022 | 6:0:0 | 6 |
| **5** | Garg and Kaur, 2020 | 6:0:0 | 6 |
| **6** | Shimotsu and Horikawa, 2016 | 6:0:0 | 6 |
| **7** | Grambal et al. 2016 | 6:0:0 | 6 |
| **8** | Hasan and Musleh, 2018 | 4:2:0 | 4 |

Appendix 4: Studies included in review and critical appraisal score.

# Appendix 5: List of the excluded studies.

|  | **Study Title and Author** | **Reason of exclusion** |
| --- | --- | --- |
| 1 | Social Anxiety and Depression Stigma Among Adolescents. (Lynch, McDonagh and Hennessy, 2021) | Study not including adult participants. Participants were less than 18 years  old. |
| 2 | Depression Severity and Depression Stigma Among Students: A Survey of Universities in Five Countries.  (Musa et al., 2020) | Study not including adult participants. Participants were less than 18 years  old. |
| 3 | Stigma about mental disease in Portuguese medical students: a cross-sectional study.  (Moreira, Oura and Santos, 2021) | Study not including adult participants. Participants were less than 18 years  old. |
| 4 | Applying Corrigan's progressive model of self-stigma to people with depression.  (Göpfert et al., 2019) | Study not measuring the impact of SSD. |
| 5 | Nurses' Attitudes Toward Psychiatric Help for Depression: The Serial Mediation Effect of Self-Stigma and Depression on Public Stigma and Attitudes Toward Psychiatric Help.  (Lee, Jeong and Yi, 2020) | The study population was nurses, not patients. |
| 6 | Depression increases subjective stigma of chronic pain. (Naushad et al., 2018) | - Study aimed to explore the stigma of depression in relation to chronic disease. - The impact of SSD   is not mentioned. |
| 7 | Stigma Towards Depression in the Workplace. (Elizabeth and Denise, 2020) | Study not including  participants with depression. |

| 8 | Continuum beliefs and the stigma of depression: An online investigation.  (Buckwitz et al., 2021) | - Study does not include participants with depression. - The study’s questioner distributed randomly   online. |
| --- | --- | --- |
| 9 | Stigma Towards Depression in Rural Ireland: A Qualitative Exploration.  (Kennedy, 2018) | Not observational study. |
| 10 | The Relation Between Mastery, Anticipated Stigma and Depression Among Older Adults in a Primary Care Setting. (Raeifar et al., 2017) | - The impact of SSD is not mentioned. - The type of stigma   is not specified. |
| 11 | Self-stigma of depression in Christians versus the general population.  (Michael and Adam, 2018) | The study does not investigate the impact of SSD. It examines the difference in experiencing SSD between two different  religious groups. |
| 12 | A Game-based school program for mental health literacy and stigma regarding depression (moving stories): protocol for a randomized controlled trial.  (Tuijnman et al., 2019) | Not observational study. |
| 13 | Depression literacy and stigma influence how parents perceive and respond to adolescent depressive symptoms.  Johnco and Rapee, 2018). | The study population was parents, not patients. |
| 14 | The Validity and the Reliability of Turkish Version of the Self-Stigma of Depression Scale.  (Kamış et al., 2019) | The study assessed the scale of SSD, not the  impact of SSD. |
| 15 | Do beliefs about depression etiologies influence the type and severity of depression stigma? The case of Arab adolescents. (Dardas et al., 2018) | Study not including adult participants. Participants were less than 18 years  old. |
| **16** | Detecting depression stigma on social media: A linguistic analysis.  (Li, Jiao and Zhu, 2018) | - Study is not concerning the self-stigma. - The study population was from the   public, not patients. |
| **17** | Personal and Perceived Depression Stigma among Arab Adolescents: Associations with Depression Severity and Personal Characteristics.  (Dardas et al., 2017) | Study not including adult participants. Participants were less than 18 years  old. |
| **18** | The experience and impact of stigma in Saudi people with a mood disorder.  (AlAteeq et al., 2018) | The type of stigma is not specified. |
| **19** | Internalized Stigma and Associated Factors among Patients with Major Depressive Disorder at the Outpatient Department of Amanuel Mental Specialized Hospital, Addis Ababa, Ethiopia, 2019: A Cross-Sectional Study.  (Alemayehu et al., 2020) | The study explores the prevalence and factors of SSD, not the impact. |
| **20** | Promoting stigma coping and empowerment in patients with  schizophrenia and depression: results of a cluster-RCT. (Gaebel et al., 2020) | Not observational study. |
| **21** | Trends in Public Stigma of Mental Illness in the US, 1996- 2018.  (Pescosolido et al., 2021) | Study is not concerning the self-stigma. |
| **22** | Stigmatizing Beliefs and Attitudes to Depression in  Adolescent School Students in Chile and Colombia. (Martínez et al., 2020) | Study not including adult participants. Participants were less than 18 years  old. |
| **23** | Self-stigma and the mediating impact of the "why try" effect on depression.  (Corrigan, Nieweglowski and Sayer, 2019). | This study examines the formation model of self- stigma and the factor associated to self-stigma,  not the impact of SSD. |
| **24** | The correlates of stigma toward mental illness among Jordanian patients with major depressive disorder.  (Rayan, Mahroum and Khasawneh, 2018). | Study is not concerning the self-stigma. |
| **25** | Internalized stigma experienced by patients with first-episode depression: A study from a tertiary care centre.  (Sahoo et al., 2018) | The study examining the prevalence of SSD, not the  impact of the SSD. |
| **26** | Internalized stigma among patients with mood disorders in Ethiopia: A cross-sectional facility-based study.  (Tesfaye et al., 2020) | The sample of the study contains patients with mood disorders, without specifying the type of the  disorder. |
| **27** | Self-Stigma Is Associated with Depression and Anxiety in a Collectivistic Context: The Adaptive Cultural Function of Self-Criticism.  (Aruta, Antazo and Paceño, 2021). | Study aimed to examine the relation between SSD and self-criticism. not the  impact of the SSD. |
| **28** | The relationship between self-stigma and depression among people with schizophrenia-spectrum disorders: A longitudinal study.  (Pellet et al., 2019) | Study included participants with more than one psychiatric  diagnosis. |
